# The Responses of Light Reaction of Photosynthesis to Dynamic Sunflecks in a Typically Shade-Tolerant Species *Panax notoginseng*

**DOI:** 10.3389/fpls.2021.718981

**Published:** 2021-10-13

**Authors:** Jin-Yan Zhang, Qiang-Hao Zhang, Sheng-Pu Shuang, Zhu Cun, Hong-Min Wu, Jun-Wen Chen

**Affiliations:** ^1^College of Agronomy and Biotechnology, Yunnan Agricultural University, Kunming, China; ^2^Key Laboratory of Medicinal Plant Biology of Yunnan Province, Yunnan Agricultural University, Kunming, China; ^3^National and Local Joint Engineering Research Center on Germplasm Innovation and Utilization of Chinese Medicinal Materials in Southwestern China, Yunnan Agricultural University, Kunming, China

**Keywords:** non-photochemical quenching, cyclic electron flow, lutein epoxide cycle, photorespiration, dynamic sunflecks, *Panax notoginseng*

## Abstract

Light is highly heterogeneous in natural conditions, and plants need to evolve a series of strategies to acclimate the dynamic light since it is immobile. The present study aimed to elucidate the response of light reaction of photosynthesis to dynamic sunflecks in a shade-tolerant species *Panax notoginseng* and to examine the regulatory mechanisms involved in an adaptation to the simulated sunflecks. When *P. notoginseng* was exposed to the simulated sunflecks, non-photochemical quenching (NPQ) increased rapidly to the maximum value. Moreover, in response to the simulated sunflecks, there was a rapid increase in light-dependent heat dissipation quantum efficiency of photosystem II (PSII) (Φ_NPQ_), while the maximum quantum yield of PSII under light (*F*_v_′/*F*_m_′) declined. The relatively high fluorescence and constitutive heat dissipation quantum efficiency of PSII (Φ_f,d_) in the plants exposed to transient high light (400, 800, and 1,600 μmol m^–2^ s^–1^) was accompanied by the low effective photochemical quantum yield of PSII (Φ_PSII_) after the dark recovery for 15 min, whereas the plants exposed to transient low light (50 μmol m^–2^ s^–1^) has been shown to lead to significant elevation in Φ_PSII_ after darkness recovery. Furthermore, PSII fluorescence and constitutive heat dissipation electron transfer rate (*J*_f,d_) was increased with the intensity of the simulated sunflecks, the residual absorbed energy used for the non-net carboxylative processes (*J*_NC_) was decreased when the response of electron transfer rate of NPQ pathway of PSII (*J*_NPQ_) to transient low light is restricted. In addition, the acceptor-side limitation of PSI [Y(NA)] was increased, while the donor-side limitation of photosystems I (PSI) [Y(ND)] was decreased at transient high light conditions accompanied with active cyclic electron flow (CEF). Meanwhile, when the leaves were exposed to transient high light, the xanthophyll cycle (V cycle) was activated and subsequently, the *J*_NPQ_ began to increase. The de-epoxidation state [(Z + A)/(V + A + Z)] was strongly correlated with NPQ in response to the sunflecks. In the present study, a rapid engagement of lutein epoxide (Lx) after the low intensity of sunfleck together with the lower NPQ contributed to an elevation in the maximum photochemical quantum efficiency of PSII under the light. The analysis based on the correlation between the CEF and electron flow devoted to Ribulose-1, 5-bisphosphate (RuBP) oxygenation (*J*_O_) indicated that at a high light intensity of sunflecks, the electron flow largely devoted to RuBP oxygenation would contribute to the operation of the CEF. Overall, photorespiration plays an important role in regulating the CEF of the shade-tolerant species, such as *P. notoginseng* in response to transient high light, whereas active Lx cycle together with the decelerated NPQ may be an effective mechanism of elevating the maximum photochemical quantum efficiency of PSII under light exposure to transient low light.

## Introduction

Light is highly heterogeneous in natural conditions since it fluctuates over short (seconds) and long (hours, days, and seasons) timescales (Townsend et al., 2017, 2018a,b). The fluctuating light usually lasts only a few seconds to minutes, but it can contribute 20–80% of the total solar energy received by the leaves (Chazdon and Pearcy, 1991). On the other hand, plants (e.g., understory species) are commonly exposed to transient high light, which can readily exceed its requirement for photosynthesis (Ruban, 2016; Townsend et al., 2018b). The excessive light energy may induce photo-inhibition of photosystem II (PSII) and even cause photo-damage to the photosynthetic apparatus, and consequently reduce the photosynthetic carbon gain (Niyogi and Truong, 2013; Vialetchabrand et al., 2017). Photoprotection in the shade-tolerant species exposed to dynamic sunflecks is particularly important as the time to reach maximum photosynthetic rate lags dramatically behind the onset of sunflecks (Way and Pearcy, 2012; Mathur et al., 2018). The model plant *Arabidopsis thaliana* might optimize electron transport and PSI photoprotection mediated by phosphorylation of vesicle-like proteins in response to the dynamic sunflecks (Tikkanen et al., 2010; Grieco et al., 2012). It is, therefore, crucial to understand the mechanism underlying photoprotection in the shade-tolerant species under dynamic light conditions, however, it receive relatively little attention over the past decades (Tikkanen et al., 2012).

Non-photochemical quenching (NPQ) is one of the most effective ways for plants to dissipate excess light energy. The mechanism of NPQ associated with heat dissipation is not well understood, but PsbS proteins and the xanthophyll cycle play an important role in regulating the NPQ processes (Hubbart et al., 2012; Ikeuchi et al., 2014). When tobacco (*Nicotiana tabacum*) and *A. thaliana* are exposed to constant high light, violaxanthin (V) is de-epoxied to form antheraxanthin (A), which is further de-epoxied to form zeaxanthin (Z) (Garcia-Molina and Leister, 2020; Tan et al., 2020). Z is used as the quenching site of excess excitation energy to dissipate excess light energy (Jahns and Holzwarth, 2012). However, in *N. tabacum* and *A. thaliana* grown under constant low light, the epoxidation of Z to V may accelerate the biomass production by increasing the photosynthetic efficiency (Cao et al., 2018; Da et al., 2018). NPQ is positively correlated with V de-epoxidation state [(Z + A)/(V + A + Z)] under steady-state light, however, it is still unknown whether the xanthophyll cycle (V cycle) is activated and it relates to NPQ under the transient high light. In addition, lutein epoxide (Lx) cycle is widely found in the shade-tolerant plants (Bungard et al., 1999; Matsubara et al., 2005; Esteban et al., 2010; Förster et al., 2011; García-Plazaola et al., 2012). It has been reported that the Lx cycle is activated to quench the excess light energy and function as a photoprotector in the understory shade plants *Virola elongata* and *Inga sapindoides* in response to the dynamic sunflecks (Matsubara et al., 2007, 2009). However, it has not been extensively studied for the presence of the Lx cycle in the typically shade-tolerant species and the role of the Lx cycle in response to different intensities of dynamic light.

Absorbed light energy is managed through several competitive pathways, such as thermal processes fluorescence, and photochemistry (Hendrickson et al., 2004; Ishida et al., 2014). NPQ-associated thermal dissipation is a photoprotection mechanism of PSII (Demmig-Adams et al., 2012; Jahns and Holzwarth, 2012). Surprisingly, only a small proportion of leaf-absorbed light energy is finally used for photosynthetic carboxylation, with most of the energy lost through the regulatory thermal dissipation and fluorescence under high light conditions (Hendrickson et al., 2004; Chen et al., 2016; Huang et al., 2019). Thus, the excitation energy use and energy allocation in PSII complexes are important for resolving the response of photosynthetic organs to the environmental factors (Hendrickson et al., 2004; Kornyeyev and Hendrickson, 2007; Chen et al., 2016). The quantum yield of dissipation associated with NPQ (Φ_NPQ_) is high and the quantum yield of electron transport in PSII (Φ_PSII_) is low in rice (*Oryza sativa*) grown under constant high light (Ishida et al., 2014), suggesting that most of the light energy absorbed by the plants is safely dissipated in the form of NPQ. However, relatively little is known about the distribution and balance of light energy absorbed by PSII in the typically shade-tolerant plants in response to different intensities of transient light.

The cyclic electron flow around photosystem I (CEF-PSI) is thought to protect plants from the damages that occurs due to the over-reduction of the thylakoids under fluctuating light (Huang et al., 2011, 2012b; Kono et al., 2014). The CEF-dependent proton gradient not only promotes ATP synthesis but also binds the heat dissipation and oxygen evolving complex to protect PSII from photo-inhibition. Furthermore, the interception of PSI photo-inhibition is mainly due to the reduction of over-reduction and superoxide anion production on the PSI receptor side by CEF (Huang et al., 2012a). CEF activates the thermal dissipation process of NPQ by inducing the formation of ΔpH (proton gradient across the thylakoid membrane), regulates the redox state of P700, and controls the electron transport by the *Cytb6f* complex (Cytochrome *b6f* complex), thereby protecting PSII and PSI from photooxidation (Miyake et al., 2005; Huang et al., 2015b,c). This has also been confirmed by the performance observed in *N. tabacum* grown under high light (Endo et al., 1999). The evidence is accumulating that mitigation of PSI donor and acceptor side photoinhibition in *Cerasus cerasoides*, *A. thaliana*, and *Bletilla striata* grown under fluctuating light could be achieved by CEF initiation (Yang et al., 2019a,b). The plants increase the oxidation of P700 to inhibit the production of reactive oxygen species (ROS) through CEF in response to high light (Miyake et al., 2005; Huang et al., 2015b). Moreover, the expression level of NDH-dependent CEF genes was elevated, suggesting that CEF activation could meet the ATP/nicotinamide adenine dinucleotide phosphate (NADPH) requirements of increased photorespiration (Tikkanen et al., 2010; Suorsa et al., 2012). Thus, there is a tight connection between the CEF and photorespiration (Foyer et al., 2012; Sunil et al., 2019). Surprisingly, little is known about the role of the photorespiratory pathway in conjunction with the CEF in adapting to different intensities of transient light in the shade plants, which are often disturbed by sunflecks.

*Panax notoginseng* (Burkill) is a perennial herb belonging to the *Araliaceae* family, which is a typically shade-tolerant species (Chen et al., 2016; Xu et al., 2018). In agricultural production, *P. notoginseng* is planted in a shaded environment constructed by the shade nets. In our previous work, 9.6–11.5% of full sunlight (FL) is preferred for the growth of *P. notoginseng* (Zuo et al., 2014; Kuang et al., 2015; Xu et al., 2018). It has been demonstrated that more electrons in the high-light-grown *P. notoginseng* illuminated by constant high light were consumed by the non-net carboxylative processes to enhance the NPQ associated with heat dissipation; correspondingly, low-light-grown *P. notoginseng* protects the photosynthetic apparatus from photo-damage by decelerating the photochemical efficiency of PSII (Chen et al., 2016). The photosynthetic rate of *P. notoginseng* responds quickly to sunflecks and the response rate is more significantly limited by stomata in our previous study (Chen et al., 2014). However, fewer studies have previously investigated the light reaction of photosynthesis in the typically shade-tolerant species *P. notoginseng exposed to* sunflecks with different intensities. In the present study, we hypothesized that: (1) the greater increase in CEF activity in response to the transient low light may accommodate the electron flows; (2) Lx cycle exist in the typically shade-tolerant species in response to the transient low light, and Lx may play the role of improving light-harvesting efficiency; (3) NPQ coupled to the de-epoxidation in the V cycle might attribute to the energy dissipation in response to the transient high light; (4) the photorespiration pathways may play a role in regulating the photosynthetic electron flow under transient high light condition.

## Materials and Methods

### The Growth Condition

The experiments were conducted at the experimental farm of Yunnan Agricultural University in Kunming, Yunnan Province, China (altitude, 1,976 m; longitude 102°45′32″, latitude 25°8′2″; annual precipitation, 1,000 mm; annual average temperature, 15.10°C). There is plenty of rain from May to October and very little rain from November to April. A shade house was constructed using a special shade net for *P. notoginseng*. Light intensity in the shade house was collected every 10 s from 6:00 to 19:00 on a clear, cloudless day using a Li-1500 (Li-Cor, NE, United States) light quantum meter. FL intensity was measured simultaneously as a control. The light transmittance of the shade house was about 10% FL, which is a suitable light intensity for *P. notoginseng* growth. The soil was local raw soil (red soil). According to the method of Abedi-Koupai et al. (2006), the physical and chemical properties of the soil were analyzed: total nitrogen (N), 2.01 g kg^–1^, total phosphorus (P), 7.273 g kg^–1^, hydrolysable N, available P, 48.39 mg kg^–1^, available K, 1910.00 mg kg^–1^, pH, 6.42, and organic matter, 57.35 g kg^–1^.

Healthy 1-year-old *P. notoginseng* was disinfected with 0.1% potassium permanganate for 5 min. In January, the seedlings were planted in white pots contained with 10 kg disinfected raw soil, and there are three seedlings in each pot. Fertilization was conducted from April to July. Compound fertilizer, monopotassium phosphate, and potassium sulfate were applied with 0.4, 0.2, and 0.3 g/pot, respectively. During the growing period, insecticides (polyoxin, chlorothalonil) were sprayed to control the disease. In August (linear phase), *P. notoginseng* was used to determine the chlorophyll fluorescence and photosynthetic pigment content.

### Chlorophyll Fluorescence

According to the method of Kramer et al. (2004), the chlorophyll fluorescence was measured using Dual-PAM-100 (Heinz Walz, Effeltrich, Germany). *P. notoginseng* was placed in an opaque incubator (MRC-1100E-LED, Gunning, Shanghai, China) for dark adaptation (more than 1 h), and the minimum fluorescence of PSII (*F*_o_) and maximum fluorescence (*F*_m_) were measured at 0 μmol m^–2^ s^–1^ light intensity. According to the previous studies, the light saturation point of *P. notoginseng* is 100–150 μmol m^–2^ s^–1^ (Chen et al., 2014). Thus, in the process of measuring the fluorescence induction curve, *P. notoginseng* was suddenly exposed to the simulated sunflecks for 30 min with the light intensity of 50 μmol m^–2^ s^–1^ (transient low light), 100 μmol m^–2^ s^–1^ (light saturation point), 400 μmol m^–2^ s^–1^ (transient moderate light), 800 μmol m^–2^ s^–1^ (transient high light), 1,600 μmol m^–2^ s^–1^ (transient high light), respectively, followed by the dark adaptation for 15 min, and the whole process lasted for 45 min.

Photosystem II chlorophyll minimum fluorescence (*F*_o_′), maximum fluorescence (*F*_m_′), and light-adapted steady-state fluorescence (*F*_s_) after light adaptation were taken two times at 30 s intervals at the beginning, then at intervals of 1 min. Five replicates were randomly selected from each treatment for the analysis of chlorophyll fluorescence. The chlorophyll fluorescence parameters of PSII are calculated as follows (Genty et al., 1989; van Kooten and Snel, 1990; Demmig et al., 1996; Hendrickson et al., 2004). Maximum photochemical quantum efficiency of PSII under light (*F*_v_′/*F*_m_′) = (*F*_m_′ − *F*_o_′)/*F*_m_; NPQ coefficient of PSII (NPQ) = (*F*_m_ − *F*_m_′)/*F*_m_′; photochemical quenching coefficient of PSII (qP) = (*F*_m_′ − *F*_s_)/(*F*_m_′ − *F*_o_′); effective quantum efficiency of PSII (Φ_PSII_) = Y(II) = (*F*_m_′ − *F*_s_)/*F*_m_′; fluorescence and constitutive heat dissipation quantum efficiency of PSII (Φ_f,d_) = Y(NO) = *F*_s_/*F*_m_; light-dependent heat dissipation quantum efficiency of PSII (Φ_NPQ_) = Y(NPQ) = *F*_s_/*F*_m_′ − *F*_s_/*F*_m_; electron transfer rate of photochemical quenching pathway of PSII (*J*_PSII_) = Φ_PSII_ × PFD × 0.84 × 0.5; fluorescence and constitutive heat dissipation electron transfer rate of PSI (*J*_f,d_) = Φ_f,d_ × PFD × 0.84 × 0.5; electron transfer rate of NPQ pathway of PSII (*J*_NPQ_) = Φ_NPQ_ × PFD × 0.84 × 0.5; the electron transfer rate of PSII [ETR(II)] = Y(II) × PFD × 0.84 × 0.5.

### Measurement of Light Absorption in Photosystem I

The light absorption of PSI was measured with reference to the measurement method of Huang et al. (2012b), and the redox state of P700 was measured using a Dual-PAM-100 measurement system (Heinz Walz, Effeltrich, Germany) with a dual wavelength (830/875 nm). After pre-illumination with far-red light, a saturating pulse of 10,000 μmol m^–2^ s^–1^ at 600 ms is applied and the signal of P700 is lowest when all of the P700 is in the reduced state. The determination of the highest P700 signal (*P*_m_) is similar to that of the maximum chlorophyll fluorescence, except that 10 s of far-red light exposure was required before the determination of the highest P700 signal. PSI reaction center P700 maximum fluorescence (*P*_m_′) was similar to *F*_m_′, except that actinic light was used instead of far-red light. The calculation of PSI parameters was referred to the method of Miyake et al. (2005). Photochemical quantum yield of PSI [Y(I)] = (*P*_m_′ − *P*)/*P*_m_; donor terminal heat dissipation efficiency of PSI [Y(ND)] = 1 − P700red = *P*/*P*_m_; receptor terminal heat dissipation efficiency of PSI [Y(NA)] = (*P*_m_ − *P*_m_′)/*P*_m_; the electron transfer rate of PSI [ETR(I)] = Y(I) × PFD × 0.84 × 0.5; cyclic electron transfer size of PSI (CEF) = *J*_PSI_ – *J*_PSII_ = ETR(I) – ETR(II).

### Steady-State Gas Exchange Measurements

The steady-state gas exchange parameters were determined using the photosynthesis system (Li-6400XT, Li-Cor, NE, United States) with the 2 cm^2^ fluorescence leaf chamber. The gas exchange parameters of healthy 2-year-old *P. notoginseng* were measured from 9 to 11 a.m. on a sunny day, with five replicates per treatment (*n* = 5). The CO_2_ content in the chamber was maintained at 400 μmol mol^–1^ during the measurement of photosynthetic light response curves. The leaf was induced under the light intensity of 500 μmol m^–2^ s^–1^ (red light: blue light = 9:1) for 10 min. The automatic measurement procedure of the instrument was started after the data of all parameters were relatively stable. The gradient of light intensity was listed in the following order: 500, 300, 200, 150, 100, 80, 60, 40, 20, 0 μmol m^–2^ s^–1^. Induction is stabilized for 120–180 s at each light intensity, during which the gas exchange parameters are collected and the data are saved after the measurement. The relationship between the net photosynthetic rate (*P*_n_) and photosynthetic photon flux density (PFD) was fitted, *P*_n_ = *P*_max_ − *P*_max_*C*_0_e^–α^ ^PFD/Pmax^ (Bassman and Zwier, 1991). In the formula, *P*_max_ is the maximum net photosynthetic rate, α is the apparent quantum efficiency (AQY), and *C*_0_ is the index where the net photosynthetic rate level off to 0 under low light. According to the parameters in the formula, the following parameters can be calculated, respectively: dark respiration rate (*R*_d_) = *P*_max_ − *P*_max_*C*_0_; light compensation point (LCP) = *P*_max_ ln(*C*_0_)/α; light saturation point (LSP) = *P*_max_ ln(100*C*_0_)/α.

The light intensity in the chamber was maintained at 500 μmol m^–2^ s^–1^ and the leaf was induced under the CO_2_ intensity of 400 μmol mol^–1^ for 10 min. The gradient of reference cell CO_2_ concentration was set in the following order: 400, 300, 250, 200, 150, 100, 50, 400, 600, 800, 1,000, 1,200, 1,500 μmol mol^–1^. According to the method of Xu (2002), a linear regression was done for the points in the CO_2_ response curve where the internal leaf CO_2_ concentrations (*C*_*i*)_ were below 250 μmol mol^–1^ to obtain the carboxylation efficiency (CE), CO_2_ compensation point (*Γ*^∗^), and to calculate the rate of photorespiration (R_*L*_) = CE × *I*^∗^. The maximum carboxylation rate (*V*_cmax_) and maximum electron transfer rate (*J*_max_) were calculated by using the method of Bernacchi et al. (2001).

Before measuring the light induction curve, *P. notoginseng* was moved into an opaque incubator (MRC-1100E-LED, Gunning, Shanghai, China) for dark adaptation, during which the plants were covered with a black bag. The CO_2_ concentration in the reference chamber was controlled to be 400 μmol mol^–1^. The leaves of *P. notoginseng* were put into the leaf chamber, and the light intensity in the leaf chamber was 0 μmol m^–2^ s^–1^. An automatic measurement was started after the data were stabilized. During measurement, the leaves were irradiated at 0 μmol m^–2^ s^–1^ light intensity for 2 min, and then induced with 400 μmol m^–2^ s^–1^ light intensity for 30 min. Data were collected every 30 s, and the gas exchange parameters were simultaneously collected.

According to the light response curve, the CO_2_ response curve, and light induction curve, the following relevant parameters can be obtained: net photosynthetic electron transfer rate (*J*_CO2_) = *P*_net_ × 4; the residual absorbed energy used for the non-net carboxylative processes *(JNC) = JPSII − JCO2* (Losciale et al., 2010). According to the method of Valentini et al. (1995), the rates of electron carboxylation (*J*_C_) and oxidation (*J*_O_) were calculated: *J*_O_ = 2/3 × [*J*_T_ – 4 × (*P*_n_ + *R*_d_)], *J*_C_ = 1/3 × [*J*_T_ + 8 × (*P*_n_ + *R*_d_)]; total photosynthetic electron flow through PSII (*J*_T_) = PFD × Y(II) × 0.84 × 0.5.

### Determination of Photosynthetic Pigment Content

*Panax notoginseng* was placed in a dark incubator (MRC-1100E-LED, Gunning, Shanghai, China) for 12 h at a temperature of 25°C. After 12 h, *P. notoginseng* was placed under the light intensity of 50, 100, 400, 800, and 1,600 μmol m^–2^ s^–1^, respectively. The process lasted 90 min, with the first 30 min being light treatment and the last 60 min being dark treatment. The leaves were collected at 0, 5, 15, 30, 35, 45, 60, and 90 min, respectively, and the cleaned and dried leaves were wrapped in tin foil and quickly stored in liquid nitrogen. Afterward, the tin foil-wrapped leaves were stored in liquid nitrogen in a sealed tin box at −80°C in the refrigerator. To measure the photosynthetic pigment content, 0.2 g leaves were weighed in the dark condition, repeatedly extracted with cold acetone, and fixed to 10 ml. According to the method of Susan and Thayer (1990) and Zhao et al. (1995) with minor modifications, the content of V, anteraxantin (A), Z, lutein (L), and Lx were determined by high-performance liquid chromatography (HPLC) (Agilent 1260, CA, United States). Then, 1.2114 g Tris was dissolved in pure water and fixed into a 100 ml volumetric flask (0.1 mol L^–1^ Tris). 0.83 ml of 36–38% HCl solution was fixed into a 100 ml volumetric flask with pure water, (0.1 mol L^–1^ HCl). Further, 50 ml of 0.1 mol L^–1^ Tris solution and 40.3 ml of 0.1 mol L^–1^ HCl solution were mixed and the pH was adjusted to 7.5 with Tris mother liquor and HCl mother liquor to obtain 0.05 mol L^–1^ Tris–HCl buffer. Mobile phase A was prepared with acetonitrile and 0.05 mol L^–1^ Tris–Hc1 buffer at 70:3; mobile phase B was prepared with methanol and n-hexane at 5:1. The extracts were filtered through a 0.22 μm pore size membrane and sampled on a ZORBAX SB-C18 (5 μm, 4.6 mm × 250 mm) column with a column temperature of 25.0°C and a flow rate of 1 ml min^–1^ at a detection wavelength of 445 nm. The standards were purchased from Sigma and ChromaDex (CA, United States) with a purity of >98%.

### Statistical Analysis

The data were counted using Microsoft Excel 2007 software (Microsoft Corp., WA, United States). The experimental site was divided into 15 plots, and 12 pots were placed per plot. We obtained five replicates (five healthy plants) randomly selected from each plot for the analysis of photosynthetic parameters. Also, the results were displayed as mean values of five independent plants. One-way ANOVA was performed using SPSS 19.0 software (IBM Corp., NY, United States), and Duncan’s test was applied for multiple comparisons of significant differences (*P* < 0.05), and the graphical data were presented as mean ± SE. GraphPad Prism 8.3.0 (CA, United States) software was used for graphing.

## Results

### Photosystem II Activity in Response to Simulated Sunflecks

**FIGURE 1:**
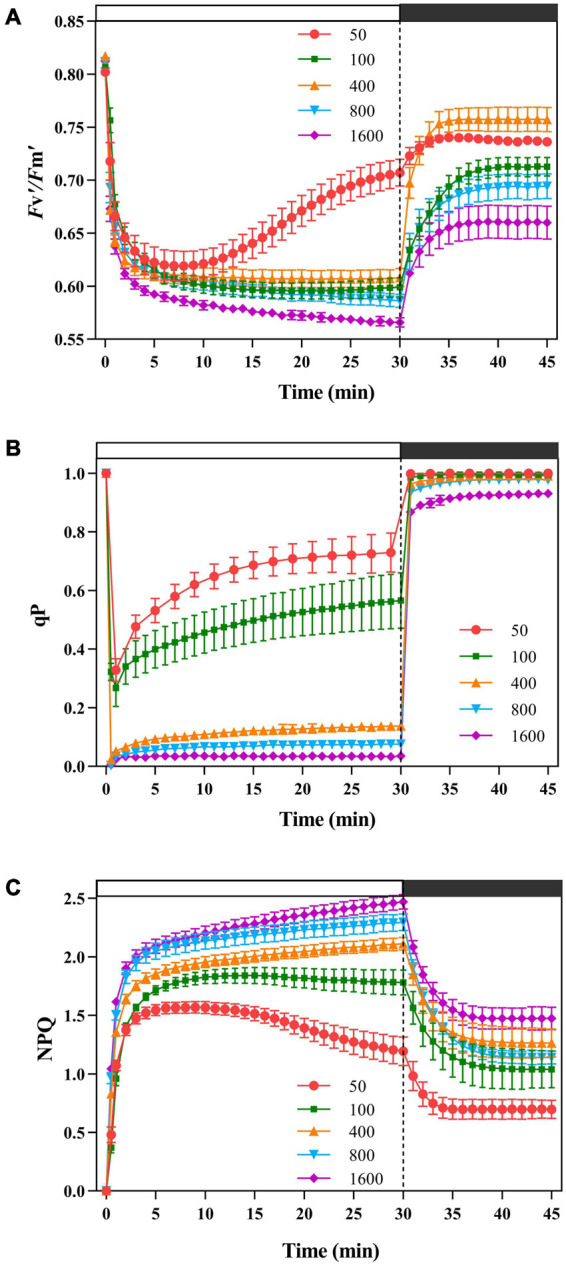
The effects of transient light on the maximum photochemical quantum efficiency of photosystem II (PSII) under light (*F*_v_′*/F*_m_′), photochemical quenching (qP), and non-photochemical quenching (NPQ). The simulated sunflecks of 50 (red), 100 (green), 400 (orange), 800 (blue), and 1,600 μmol m^–2^ s^–1^ (purple) was conducted for 30 min, respectively (the left of the dotted line), and then dark recovery was conducted for 15 min (the right of the dotted line). The *F*_v_′*/F*_m_′ **(A)**, qP **(B),** and NPQ**(C)** were recorded every minute, the value is the means ± SE (*n* = 5).

The leaf exhibited a significant difference in PSII activity in response to the simulated sunflecks (Figure 1). The *F*_v_′/*F*_m_′ decreased rapidly at different intensities of transient light. In the process of dark recovery, the individuals exposed to transient light (50 and 400 μmol m^–2^ s^–1^) recovered to the higher levels with respect to *F*_v_′/*F*_m_′, and the others recovered to lower level. These results indicated that the transient high light leaded to lower PSII activity.

### Energy Dissipation Through Non-photochemical Quenching in Response to Transient High Light

Non-photochemical quenching responded quickly and increased rapidly at different transient light (Figure 1C). The plants exposed to the transient low light (50 μmol m^–2^ s^–1^) contributed most of the energy dissipation to the qP pathway (Figure 1B). When the plants were exposed to the transient light (below 400 μmol m^–2^ s^–1^), the plants dissipated more energy through NPQ in the early stages (Figure 1C) and increased their ability to dissipate energy through photochemical quenching in the later stages (Figure 1C), while at 800 and 1,600 μmol m^–2^ s^–1^ transient light, the energy dissipation through NPQ was dominant (Figure 1C). During the dark recovery period, qP recovered rapidly at all transient light, with no significant differences between the transient light; while NPQ remained at a high level at transient high light (400, 800, and 1,600 μmol m^–2^ s^–1^) (Figure 1).

### Heat Dissipation in Response to Transient High Light

**FIGURE 2:**
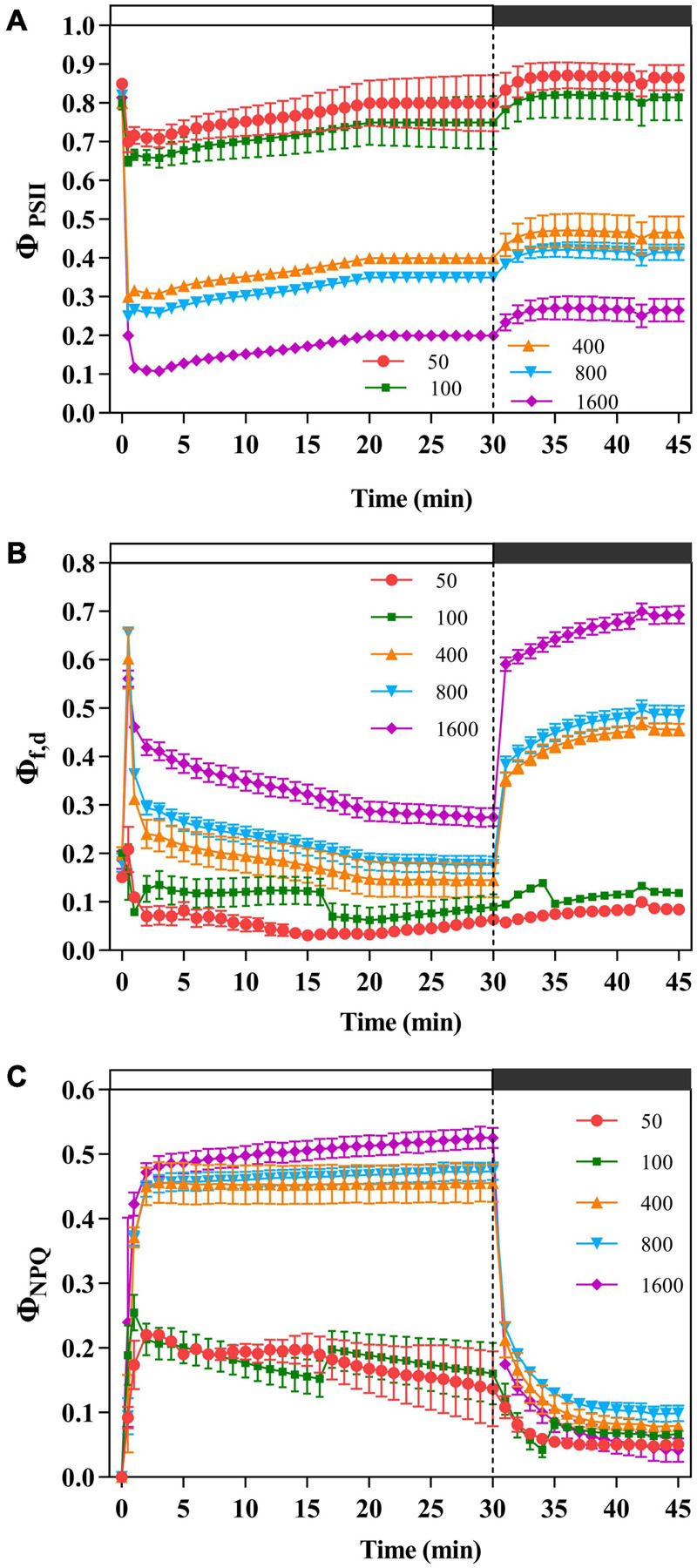
The effects of transient light on the effective photochemical quantum yield of PSII (Φ_PSII_), fluorescence and constitutive heat dissipation quantum efficiency of PSII (Φ_f,d_), and light-dependent heat dissipation quantum efficiency of PSII (Φ_NPQ_). The *Panax notoginseng* was induced for 30 min in the simulated dynamic light of 50, 100, 400, 800, and 1,600 μmol m^–2^ s^–1^, respectively (the left of the dotted line), and then dark recovery was conducted for 15 min (the right of the dotted line). Φ_PSII_ **(A)**, Φ_f_,_d_ **(B),** and Φ_NPQ_ **(C)** were recorded every minute, the value is the means ± SE (*n* = 5).

The plants were exposed to 50 μmol m^–2^ s^–1^ transient low light, the light energy received by PSII was mainly allocated to the photochemical reaction pathway (Figure 2A), while the others dissipated light energy *via* NPQ pathway (Figure 2C) and the fluorescence dissipation pathway (Figure 2B). When *P. notoginseng* was transformed from a dark environment to transient light, Φ_PSII_ decreases with the increase of transient light (Figure 2A). As the simulated sunfleck proceeded, Φ_PSII_ continuously dropped and Φ_NPQ_ slowly increased under the transient light intensity of 400, 800, and 1,600 μmol m^–2^ s^–1^ (Figures 2A,C), indicating a slow reopening of available reaction centers. Φ_f,d_ at the simulated sunflecks of 400, 800, and 1,600 μmol m^–2^ s^–1^ was approximately higher than that at 100 μmol m^–2^ s^–1^. Furthermore, when *P. notoginseng* was switched from the simulated sunflecks to a dark environment, with the increase of transient light, the extent of recovery in Φ_PSII_ decreased (Figure 2A).

### The Oxidation State of Photosystem I (P700^+^)

**FIGURE 3:**
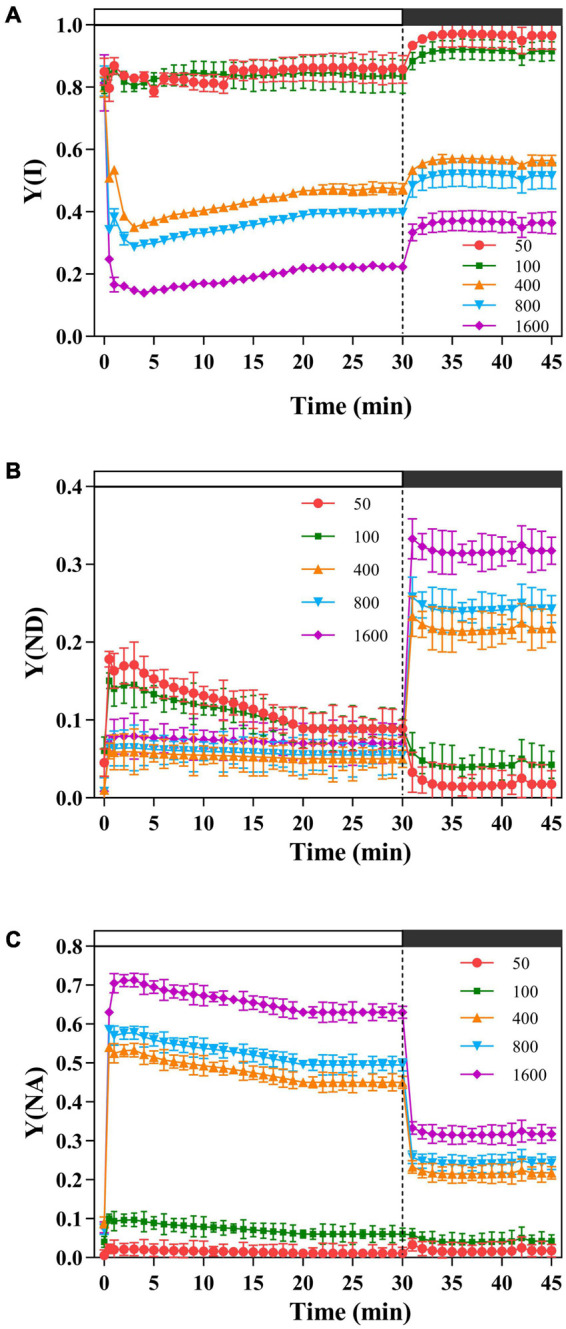
The effects of transient light on the quantum yield of photosystem I (PSI) [Y(I)], the donor-side limitation of PSI [Y(ND)], and the acceptor-side limitation of PSI [Y(NA)]. The *P. notoginseng* was induced for 30 min in the simulated dynamic light of 50, 100, 400, 800, and 1,600 μmol m^–2^ s^–1^, respectively (the left of the dotted line), and then dark recovery was conducted for 15 min (the right of the dotted line). [Y(I)] **(A)**, [Y(ND)] **(B),** and [Y(NA)] **(C)** were recorded every min, the value is the means ± SE (*n* = 5).

The light energy received by the PSI is mainly allocated to the PSI acceptor side for thermal dissipation [Y(NA)], except for the transient light of 50 and 100 μmol m^–2^ s^–1^, while the thermal dissipation at the PSI donor side [Y(ND)] is low, and the effective quantum efficiency of the PSI [Y(I)] decreases with the increase of transient light (Figure 3). When *P. notoginseng* was suddenly exposed to the simulated sunflecks, Y(ND) increased rapidly; while during darkness recovery, Y(ND) was close to the initial level (Figure 3B). Meanwhile, Y(I) decreased rapidly and then increased at transient light of 50, 100, and 400 μmol m^–2^ s^–1^ (Figure 3A). In contrast, Y(I) declined at transient light of 800, and 1,600 μmol m^–2^ s^–1^, and Y(I) rose rapidly during darkness recovery (Figure 3A).

### Cyclic Electron Flow and Electron Low Devoted to RuBP Oxygenation

**FIGURE 4:**
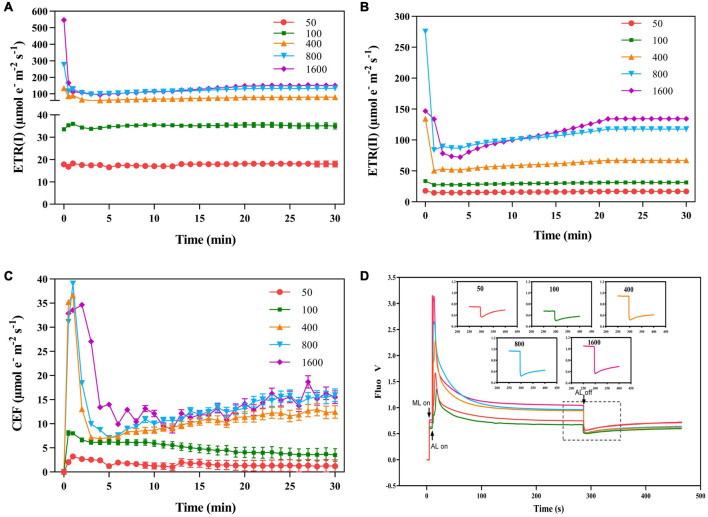
The effects of transient light on the electron transport rate of PS I [ETR(I)], electron transport rate of PSII [ETR(II)], and cyclic electron flow of PSI (CEF). The *P. notoginseng* was induced for 30 min in the simulated dynamic light of 50, 100, 400, 800, and 1,600 μmol m^–2^ s^–1^, respectively (the left of the dotted line), and then dark recovery was conducted for 15 min (the right of the dotted line). [ETR(I)] **(A)**, [ETR(II)] **(B),** and (CEF) **(C)** were recorded every min, the value is the means ± SE (*n* = 5). **(D)** Effects of transient light on post-illumination. Vertical bars indicate the timing of on (upward) and off (downward) points of measuring light (ML), actinic light (AL) of 50 μmol m^–2^ s^–1^, and a saturating light flash (SF, 12,000 μmol m^–2^ s^–1^, 800 ms), respectively. The insets are magnified traces from the boxed area of the transient increase in chlorophyll fluorescence due to NDH activity on different light intensities for 280 s.

Electron transfer rate II [ETR(II)] increased rapidly when plants were exposed to transient high light (Figure 4B). The ETR(I) is at a low level at t transient light of 50 μmol m^–2^ s^–1^ (Figure 4A). The change of CEF was similar to that of ETR(I). In a short time, the transient high light excited high CEF to protect the photosystem from damage (Figure 4C). When plants in darkness were suddenly exposed to transient light, chlorophyll fluorescence rapidly reached a maximum in a short period of time and gradually stabilized over time (Figure 4D). The photochemical light was turned off, transient light resulted in different cyclic electron transfer activities. The cyclic electron transfer activity was 50 μmol m^–2^ s^–1^ (0.0027) > 1,600 μmol m^–2^ s^–1^ (0.0024) > 800 μmol m^–2^ s^–1^ (0.0023) > 400 μmol m^–2^ s^–1^ (0.0021) > 100 μmol m^–2^ s^–1^ (0.0014), suggesting that the high cyclic electron transfer activity plays an important role in photoprotection.

**FIGURE 5:**
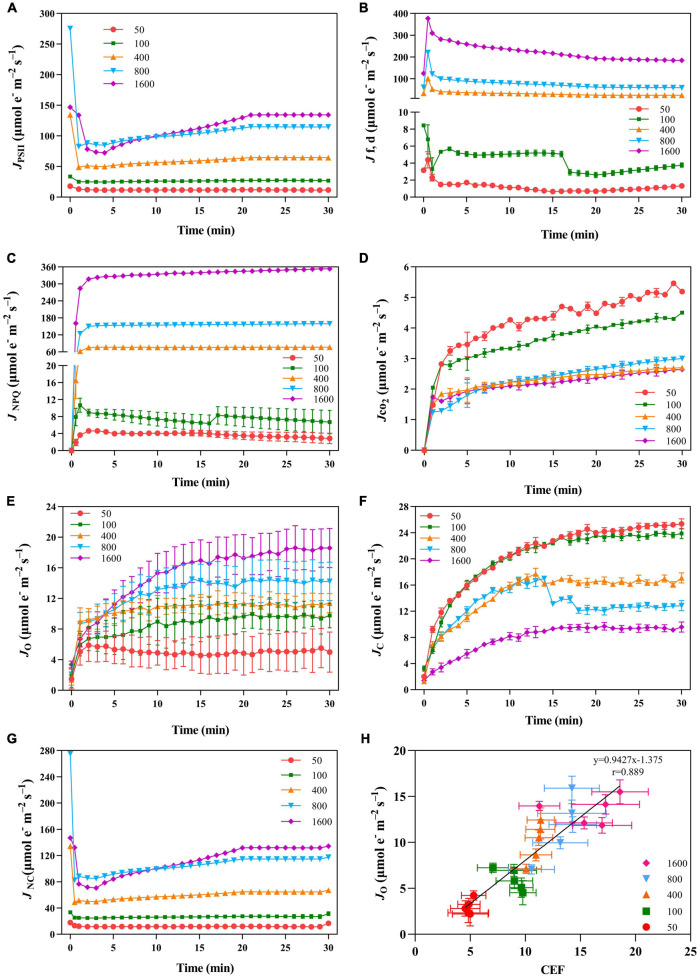
The effects of transient light on PSII photochemical quenching pathway ETR (*J*_PSII_, **A**), PSII fluorescence and constitutive heat dissipation ETR (*J*_f,d_, **B**), PSII non-photochemical quenching electron transfer rate (*J*_NPQ_, **C**), net photosynthetic electron transport rate (*J*_CO2_, **D**), rate of electron transport for oxidation reaction (*J*_O_, **E**), carboxylation reaction (*J*_C_, **F**), and the residual absorbed energy used for the non-net carboxylative processes (*J*_NC_, **G**). **(H)** Correlation between CEF and *J*_O_ under different transient light intensities. *J*_PSII_, *J*_NPQ_, *J*_f,d_, *J*_CO2_, *J*_O_, and *J*c were recorded every minute, the value is the means ± SE (*n* = 5).

The value of *J*_PSII_ increased rapidly when *P. notoginseng* was suddenly exposed to transient light (Figure 5A). A larger value of *J*_PSII_ was exhibited at transient light of 1,600 μmol m^–2^ s^–1^ and a smaller value of *J*_PSII_ was exhibited at transient low light (Figure 5A). There was a significant difference in *J*_NPQ_ under the different levels of transient light (Figure 5C), indicating that the electron transfer rate through the NPQ of PSII is mainly influenced by the light intensity. The plants were suddenly exposed to transient light, *J*_f,d_ rises for a short time and falls over time. The value of *J*_f,d_ was increased with the intensity of the simulated sunflecks as presented by the elevation of Φ_f,d_ at the simulated sunflecks of 400, 800, and 1,600 μmol m^–2^ s^–1^ (Figures 2B, 5B). Moreover, the maximum values of *J*_CO2_ and *J*_C_ were generally recorded in the transient low light (Figures 5D,F). At the high light intensity of sunflecks, a decrease in the electron flow devoted to Ribulose-1, 5-bisphosphate (RuBP) carboxylation indicated the reduction in the incident chloroplast CO_2_ concentration (Figure 5F). Electron flow was largely devoted to RuBP oxygenation in response to the high light intensity of sunflecks (Figure 5E). Based on the correlation between CEF and electron flow devoted to RuBP oxygenation (*J*_O_), the electron flow that was largely devoted to RuBP oxygenation would contribute to the operation of the CEF (Figure 5H).

### Non-photochemical Quenching Attached to Xanthophyll Cycle

**FIGURE 6:**
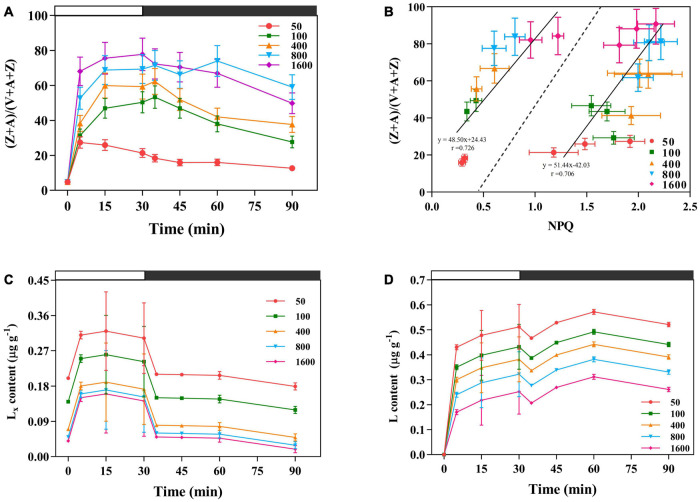
The effects of transient light on xanthophylls de-epoxidation state xanthophyll cycle (V cycle) **(A)**. V is violaxanthin, A is anteraxantin, Z is zeaxanthin. **(B)** Correlation between NPQ and V cycle under different transient light intensities. (Z + A)/(V + A + Z) is the xanthophylls de-epoxidation state. The data to the left of the dashed line are the NPQ and (Z + A)/(V + A + Z) obtained at different transient light intensities for 35 and 45 min, respectively. The data to the right of the dashed line are the NPQ and (Z + A)/(V + A + Z) obtained at different transient light intensities for 5, 15, and 30 min, respectively. **(C,D)** The effects of transient light on the lutein epoxide (Lx) cycle. Lx: lutein epoxide, L: lutein (L), the amount of Lx de-epoxidized to L. The dark-adapted lutein level (time 0) was subtracted from the lutein content measured during the experiment. The value is the means ± SE (*n* = 5).

The xanthophylls de-epoxidation state (Z + A)/(V + A + Z) was higher in the plants exposed to transient high light (Figure 6A). The NPQ and V de-epoxidation state also showed a significant linear positive correlation (Figure 6B). On the other hand, under the transient high light conditions, the amount of Lx de-epoxidized to L was relatively reduced (Figure 6D), rapid engagement of Lx content after the low intensity of sunfleck contributes together with the lower NPQ to an elevation in the maximum photochemical quantum efficiency of PSII under light (Figures 1A,C, 6C).

### Non-photochemical Quenching in Response to Simulated Fluctuating Light

**FIGURE 7:**
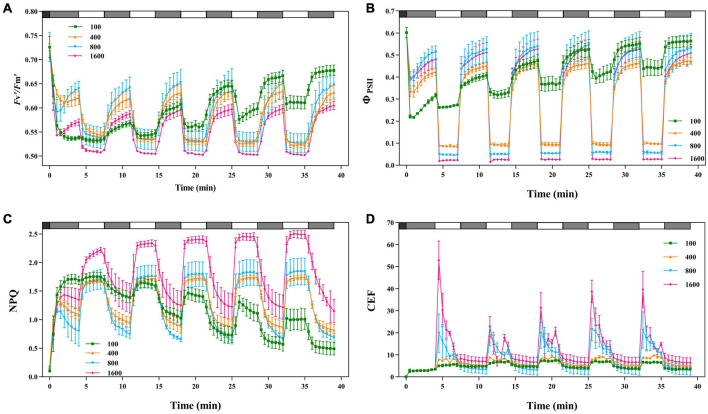
The effects of fluctuating light on *F*_v_′*/F*_m_′, Φ_PSII_, NPQ, and CEF. The black bars indicate dark, the gray bars indicate low light (50 μmol m^–2^ s^–1^) for 4 min and the white bars indicate transient light (100, 400, 800, and 1,600 μmol m^–2^ s^–1^) for 3 min. *F*_v_′*/F*_m_′ **(A)**, Φ_PSII_ **(B)**, NPQ**(C),** and CEF **(D)** were recorded every 30 s, the value is the means ± SE (*n* = 5).

In the simulated sunflecks environment with alternating fluctuations between 4 min low light (50 μmol m^–2^ s^–1^) and 3 min high light (100, 400, 800, and 1,600 μmol m^–2⋅^s^–1^), *F*_v_′/*F*_m_′ and Φ_PSII_ gradually increased with the extension of alternating time under the light intensity of 100 μmol m^–2^ s^–1^ (Figures 7A,B). *F*_v_′/*F*_m_′ in high light (800 and 1,600 μmol m^–2^ s^–1^) did not change significantly in the alternation of fluctuating light, but *F*_v_′/*F*_m_′ in low light gradually increased with the alternation of fluctuating light Φ_PSII_, indicating that the fluctuating light has an important effect on the recovery of PSII activity in the plants. Moreover, during alternating fluctuating light, Φ_PSII_ at the light intensities of 400, 800, and 1,600 μmol m^–2^ s^–1^ was at a low level. The increase in Φ_PSII_ becomes greater when the plants were transferring from high light (800 and 1,600 μmol m^–2^ s^–1^) to low light (50 μmol m^–2^ s^–1^), suggesting that in the process of alternating fluctuations, Φ_PSII_ was inhibited under high light, and Φ_PSII_ is recovered and electron-replenished under low light.

When *P. notoginseng* in darkness was transferred to 4 min of low light (50 μmol m^–2^ s^–1^), the NPQ gradually decreased, followed by a rapid increase when *P. notoginseng* was exposed to high light (800 and 1,600 μmol m^–2^ s^–1^; Figure 7C). As the alternating fluctuating light continued, NPQ was greater at light intensities of 400, 800, and 1,600 μmol m^–2^ s^–1^, and NPQ was smaller at light intensities of 100 μmol m^–2^ s^–1^. NPQ had “memory” in response to the simulated fluctuating light. When the plants were switched from darkness to 4 min of low light (50 μmol m^–2^ s^–1^), CEF was activated (Figure 7D). The CEF was greatly excited when *P. notoginseng* was exposed to high light (800 and 1,600 μmol m^–2^ s^–1^), but gradually decreased over time. CEF at light intensities of 100 and 400 μmol m^–2^ s^–1^ increased over time.

## Discussion

Light is the ultimate resource for photosynthesis, and its intensity and spectral composition considerably vary in nature. These traits compel plants to maintain the balance between light absorption needed for photosynthesis and excess light dissipation for photo-protection (Wagner et al., 2006). Excess light may adversely affect the plants. If the photochemical capacity exceeds the input energy, the balance of energy absorption/utilization cannot be achieved (Jin et al., 2016; Crisp et al., 2017). This process leads to photo-inhibition and/or irreversible photo-oxidative damage due to the presence of ROS (Krieger-Liszkay et al., 2008; Szymańska et al., 2017). The plants evolve various mechanisms to adjust their response to dynamic sunflecks. Some mechanisms occur very quickly, such as NPQ (Carraretto et al., 2016) and cycle electron flow (Yamori et al., 2016; Kono et al., 2017, 2019).

### Energy Dissipation Through Non-photochemical Quenching Was Dominant in Response to Transient High Light

Non-photochemical quenching responds very rapidly to the sunflecks, which have been documented in the tropical rainforest plants (Maxwell and Johnson, 2000; Tausz et al., 2005). The plants were exposed to the simulated sunflecks, NPQ increased rapidly to the maximum (Figure 1C). Moreover, in response to the simulated sunflecks, there was also a rapid increase in Φ_NPQ_ (Figure 2C), while the *F*_v_′/*F*_m_′ declined (Figure 1A), confirming the previous studies (Tausz et al., 2005). Meanwhile, the plants exposed to the transient high light appeared to increase more rapidly and largely in NPQ than the ones exposed to the transient low light (Figure 1C), suggesting the importance of the NPQ upon exposure to the transient high light. Energy quenching (qE) characterized as a dominant part of thermal dissipation is caused by the excess light-induced proton gradients (ΔpH) across the vesicle-like membrane (Müller et al., 2001; Cazzaniga et al., 2013), and PSII subunit S (PsbS) is involved in this quenching process (Niyogi et al., 2005; Pawlak et al., 2020). However, drastic changes in the qE process are induced depending on the amount of PsbS (Johnson and Ruban, 2010, 2011). This suggests that PsbS may result in the acceleration of NPQ induction to adapt to transient light. On the other hand, *F*_v_′/*F*_m_′ and qP increased significantly while NPQ diminished after the light-to-dark transition (Figure 1), however, *F*_v_′/*F*_m_′ was difficult to return to the initial state with the increase of transient light intensity, indicating an impaired PSII (Figure 1A). A possible explanation is that the relaxation rate of NPQ lags the induction rate and is exacerbated by prolonged exposure to the excessive light conditions (Pérez-Bueno et al., 2008) so that NPQ inhibits photosynthetic quantum yield after the light-to-dark transition (Murchie and Niyogi, 2011). Interestingly, the recovery of *F*_v_′/*F*_m_′ was more pronounced at transient light of 400 μmol m^–2^ s^–1^ than at transient light intensity of 100 μmol m^–2^ s^–1^, which may be due to the higher CEF activity of the PSI at transient moderate light, where light energy mediates a balance between the photo-protection and light energy utilization (Figures 1A, 4D; Sato et al., 2014; Kromdijk et al., 2016).

### The Greater Increase in Cyclic Electron Flow Activity in Responses to Transient Low Light May Accommodate the Electron Flows

The value of Φ_f,d_ at the simulated sunflecks of 400, 800, and 1,600 μmol m^–2^ s^–1^ was higher than that at 100 μmol m^–2^ s^–1^ (Figure 2B). A photo-damage was observed in *A. thaliana* exposed to transient high light (Li et al., 2002; Ikeuchi et al., 2014). Thus, the relatively high Φ_f,d_ in plants exposed to the transient high light (400, 800, and 1,600 μmol m^–2^ s^–1^) can be explained by a relatively high photo-damage as estimated from the low Φ_PSII_ after dark recovery for 15 min (Figures 2A,B). The photo-damage rate is positively correlated with light intensity (Allakhverdiev and Murata, 2004), and the repair rate of the PSII depends on the intensity of the incident light, but it is maximized at relatively low light intensities (Takahashi and Murata, 2008). This interpretation was verified in the present study that the plants exposed to the simulated sunflecks of 50 μmol m^–2^ s^–1^ have been shown to lead to significant elevation in Φ_PSII_ after darkness recovery, indicating a healthy PSII. A similar result was also shown when *Haberlea rhodopensis* was subjected to weak light (Durgud et al., 2018). When *P. notoginseng* was exposed to the simulated sunflecks close to the light saturation point, the light energy absorbed by the plants coincided with the need for photosynthesis, so that the value of Φ_PSII_ significantly increased after darkness recovery (Figure 2A). The photochemical quantum yield of PSI [Y(I)] decreased more drastically after the start of simulated sunflecks of 400, 800, and 1,600 μmol m^–2^ s^–1^ (Figure 3A), it should be noted that this was the result of transient high light. This was also supported by the fact that the impairment on PSI was presented in *A. thaliana* exposed to high light (Munekage et al., 2002, 2008). In this study, the simulated sunflecks of 400, 800, and 1,600 μmol m^–2^ s^–1^ increased Y(NA), while Y(ND) was decreased in *P. notoginseng* (Figures 3B,C), this indicated that when being exposed to transient high light, the acceptor-side reactions restrict PSI activity. When the plants are suddenly exposed to the transient light above their light saturation point, the acidification of the thylakoid lumen is not able to sufficiently downregulate the linear electron flow, and the relaxed state of the thylakoid causes an electron rush to PSI, which leads to a reduction in the electron acceptors, oxygen photoreduction, and ROS formation (Tikkanen et al., 2010; Kono et al., 2014; Wang et al., 2015). By contrast, Y(NA) of PSI is decreased while Y(ND) is increased in *P. notoginseng* exposed to the simulated sunflecks of 50 μmol m^–2^ s^–1^ (Figures 3B,C), suggesting that the enhanced oxidation state of PSI (P700^+^) is also a mechanism to ensure the integrity of PSI. PSI photo-inhibition with large differences between the transient high light and transient low light can be explained by the photosynthetic alternative electron flows interacting with PSI. The greater increase in CEF activity in responses to the transient low light may accommodate the electron flows, consequently avoiding the response of photo-oxidative damage (Figure 4D; Kono et al., 2014).

### Non-photochemical Quenching Coupled to the De-epoxidation in the V Cycle Might Attribute to Energy Dissipation in Response to Transient High Light

Photosystem II fluorescence and constitutive heat dissipation electron transfer rate (*J*_f,d_) was increased with the intensity of the simulated sunflecks as presented by the elevation of Φ_f,d_ at simulated sunflecks of 400, 800, and 1,600 μmol m^–2^ s^–1^ (Figures 2B, 5B). This increased thermal and fluorescence dissipation at the high intensity of the simulated sunflecks may be the result of an imbalance in the exciton-radical pairs and relatively rapid electron transfer from the pheophytin to the primary quinone acceptor (Losciale et al., 2008). The residual absorbed energy used for non-net carboxylative processes (*J*_NC_) predominated at the simulated sunflecks of 1,600 μmol m^–2^ s^–1^ (Figure 5G). Even though *J*_NC_ usually confers more potentially detrimental effects than NPQ because of the generation of ROS during non-net carboxylative processes, these mechanisms are preferentially activated when the response of *J*_NPQ_ to high light is elevated (Losciale et al., 2008; Ishida et al., 2014). The *trans*-thylakoid pH-dependent V cycle is the main mechanism driving the heat dissipation operation, with a lumen pH (5.7–7.5) enabling optimal V de-epoxidase activity (Takizawa et al., 2007). The present study clearly showed that the simulated sunflecks of 50 μmol m^–2^ s^–1^ suppressed the (Z + A)/(V + A + Z) (Figure 6A), this could be explained by the fact that the acidity of thylakoid lumen does not activate the violaxanthin de-epoxidase (VDE) sufficiently at low intensity of sunflecks, so the plants dissipate the excess energy by alternative ways. It has been reported that the cyclic transport around PSI can create an optimal pH for VDE by elevating the *trans*-thylakoid pH (Cruz et al., 2005), CEF was increased in the plants exposed to the transient high light (400, 800, and 1,600 μmol m^–2^ s^–1^) (Figure 4C), the optimum lumen pH may activate the V cycle and subsequently the *J*_NPQ_ begins to increase (Figures 5C, 6).

### Lx Cycle Exists in the Typically Shade-Tolerant Species in Response to Transient Low Light

The operation of the Lx cycle is an additional mechanism to regulate light harvesting by antenna complex, allowing leaves with high (Z + A)/(V + A + Z) to exhibit rapid and enhanced NPQ (Matsubara et al., 2008; Esteban et al., 2010). Under the transient high light conditions, the amount of Lx de-epoxidized to L was relatively reduced (Figure 6D). A similar result was found in the earlier studies, where shaded young avocado leaves showed a decreasing trend in the total L pool after sudden exposure to sunlight, despite the deep oxidation of Lx, which may be related to photo-oxidation of L (Förster et al., 2009, 2011). The slow operation of the Lx cycle in some species may “lock in” photoprotection (Matsubara et al., 2005; Förster et al., 2011). The sudden exposure of *P. notoginseng* to transient high light was accompanied by a slow operation of the Lx cycle, therefore, based on the correlation between NPQ and the degree of de-epoxidation in the V cycle, the de-epoxidation in the V cycle might primarily attribute to energy dissipation (Figure 6B; Esteban et al., 2007; García-Plazaola et al., 2007). In particular, Lx plays a central role in light-harvesting in response to low light. Recombinant *Lhcb5* reconstituted with Lx has a high fluorescence yield (Matsubara et al., 2007). Reduced Lx content results in a slower reduction in PSII electron acceptor and rapid formation of NPQ (García-Plazaola et al., 2012). In the present study, a rapid engagement of Lx content after the low intensity of sunfleck contributes together with the lower NPQ to an elevation in the maximum photochemical quantum efficiency of PSII under light (Figures 1A,C, 6C). The present study combined with the results of previous studies suggests that the presence of Lx in the transient low light may improve the maximum photochemical quantum efficiency of PSII under the light.

### Photorespiration Plays a Role in Regulating the Cyclic Electron Flow in Response to Transient High Light

The regulation of photosynthetic electron flow in *A. thaliana* under the simulated sunflecks has been preliminarily investigated by previous work on CEF and O_2_-dependent alternative electron sinks (Kono et al., 2014; Allahverdiyeva et al., 2015; Tiwari et al., 2016). The adaptation of PSI to the sunflecks is mainly due to CEF, while photorespiration in *Arabidopsis* leaves contributes little to the reduction of photo-damage under the low intensity of sunflecks (Suorsa et al., 2012; Kono et al., 2014). The previous study with *N. tabacum* has demonstrated that the light intensity facilitates the capacity of the photorespiratory pathway, and high-light-grown leaves regulate the CO_2_ uptake and photosynthetic electron flow through the active photorespiratory pathway (Huang et al., 2014, 2015a). However, the importance of photorespiration exerted by the shade-growth plants suddenly exposed to sunflecks is unclear. In the present study, at the high light intensity of sunflecks, a decrease in the electron flow devoted to RuBP carboxylation indicated the reduction in the incident chloroplast CO_2_ concentration (Figure 5F). *A. thaliana* produces a photorespiratory response when moving from an environment with high CO_2_ concentration to ambient air, due to the reduced availability of CO_2_ (Foyer et al., 2012; Eisenhut et al., 2017), the fact is confirmed by the present study that electron flow was largely devoted to RuBP oxygenation in response to the high light intensity of sunflecks (Figure 5E). Diminished glycine/serine (Gly/Ser) in *Arabidopsis* CEF mutants grown under high light conditions indicates impaired photorespiration, suggesting a strong link between CEF and photorespiration (Florez-Sarasa et al., 2016). Similarly, based on the correlation between CEF and electron flow devoted to RuBP oxygenation (*J*_O_), the electron flow that was largely devoted to RuBP oxygenation would contribute to the operation of the CEF (Figure 5H; von Caemmerer, 2020; Marçal et al., 2021). Thus, for plants growing in the shade environment, the photorespiration pathway regulates the photosynthetic electron flow under transient high light.

## Conclusion

Energy dissipation through NPQ predominates in response to the dynamic sunflecks, the V cycle plays an important role in regulating the NPQ processes, leading to the dissipation of excess light energy. Meanwhile, CEF was highly stimulated to protect PSI from photo-inhibition in response to the dynamic sunflecks. Additionally, photorespiration plays a role in regulating the CEF in response to the transient high light, whereas the Lx cycle together with the decelerated NPQ may be an effective mechanism of elevating the maximum photochemical quantum efficiency of PSII under the light in response to the transient low light. Overall, our results indicate that NPQ and CEF may protect the photosynthesis apparatus from dynamic sunfleck in a typically shade-tolerant species.

## Data Availability Statement

The original contributions presented in the study are included in the article/supplementary material, further inquiries can be directed to the corresponding author.

## Author Contributions

J-WC directed the whole process of the experiment and made suggestions for the writing of the manuscript. J-YZ participated in the whole experiment, analyzed the relevant experimental data, and wrote the manuscript. Q-HZ and S-PS measured the light absorption in photosystem I and chlorophyll fluorescence. ZC and H-MW participated in the determination of photosynthetic pigment content and steady-state gas exchange measurements. All authors contributed to the article and approved the submitted version.

## Conflict of Interest

The authors declare that the research was conducted in the absence of any commercial or financial relationships that could be construed as a potential conflict of interest.

## Publisher’s Note

All claims expressed in this article are solely those of the authors and do not necessarily represent those of their affiliated organizations, or those of the publisher, the editors and the reviewers. Any product that may be evaluated in this article, or claim that may be made by its manufacturer, is not guaranteed or endorsed by the publisher.

## Abbreviations

CEF: cyclic electron flow
ETR(I): electron transport rate of PSI
ETR(II): electron transport rate of PSII
*F*_m_: maximum fluorescence after dark-adaptation
*F*_m_′: maximum fluorescence after light adaptation
*F*_o_: minimum fluorescence after dark-adaptation
*F*_o_′: minimum fluorescence after light adaptation
*F*_s_: light-adapted steady-state fluorescence
*F*_v_′/*F*_m_′: maximum photochemical quantum efficiency of PSII under light
*J*_f,d_: PSII fluorescence and constitutive heat dissipation electron transfer rate
*J*_NC_: the residual absorbed energy used for non-net carboxylative processes
*J*_NPQ_: electron transfer rate of non-photochemical quenching pathway of PSII
*J*_O_: electron flow devoted to RuBP oxygenation
Lx: lutein epoxide
NPQ: non-photochemical quenching
[(Z + A)/(V + A + Z)]: the de-epoxidation state
Y(I): quantum yield of PSI
Y(II): effective quantum yield of PSII photochemistry
Y(NA): the acceptor-side limitation of PSI
Y(ND): the donor-side limitation of PSI
Φ_f,d_: the relatively high fluorescence and constitutive heat dissipation quantum efficiency of PSII
Φ_NPQ_: light-dependent heat dissipation quantum efficiency of PSII
Φ_PSII_: effective photochemical quantum yield of PSII.
